# Spoligotype-specific risk of finding lesions in tissues from cattle infected by *Mycobacterium bovis*

**DOI:** 10.1186/s12917-021-02848-3

**Published:** 2021-04-07

**Authors:** Alberto Gomez-Buendia, Beatriz Romero, Javier Bezos, Francisco Lozano, Lucía de Juan, Julio Alvarez

**Affiliations:** 1 VISAVET Health Surveillance Centre, Universidad Complutense de Madrid, Madrid, Spain; 2 Departamento de Sanidad Animal, Facultad de Veterinaria, Universidad Complutense de Madrid, Madrid, Spain

**Keywords:** Animal tuberculosis, *Mycobacterium bovis*, Spoligotype, Lesion

## Abstract

**Background:**

Although the pathogenic effect of members of the *Mycobacterium tuberculosis* complex in susceptible hosts is well known, differences in clinical signs and pathological findings observed in infected animals have been reported, likely due to a combination of host and pathogen-related factors. Here, we investigated whether *Mycobacterium bovis* strains belonging to different spoligotypes were associated with a higher risk of occurrence of visible/more severe lesions in target organs (lungs and/or lymph nodes) from infected animals. A large collection of 8889 samples belonging to cattle were classified depending on the presence/absence of tuberculosis-like lesions and its degree of severity. All samples were subjected to culture irrespective of the presence of lesions, and isolates retrieved were identified and subjected to spoligotyping. The association between the presence/severity of the lesions and the isolation of strains from a given spoligotype was assessed using non-parametric tests and Bayesian mixed multivariable logistic regression models that accounted for origin (region and herd) effects.

**Results:**

Results suggested a difference in severity in lesioned samples depending on the strain’s spoligotype. An association between specific spoligotypes and presence of lesions was observed, with a higher risk of finding lesions in animals infected with strains with spoligotypes SB0120, SB0295 and SB1142 compared with SB0121, and in those coming from certain regions in Spain.

**Conclusions:**

Our results suggest that strains belonging to certain spoligotypes may be associated with a higher probability in the occurrence of gross/macroscopic lesions in infected cattle, although these observational findings should be confirmed in further studies that allow accounting for the effect of other possible confounders not considered here, and ultimately through experimental studies.

**Supplementary Information:**

The online version contains supplementary material available at 10.1186/s12917-021-02848-3.

## Background

Animal tuberculosis (TB) is a multi-host chronic-debilitating infectious disease caused by members of the *Mycobacterium tuberculosis* complex (MTC). Even though it is most commonly found in cattle, its causative agents (*M. bovis* and to a lesser extent *M. caprae*) are capable of infecting a wide spectrum of species [1, 2], including domestic species such as goat, pig and sheep, as well as wild species (e.g., wild boar, deer and badger) that can act as reservoirs and hamper efforts invested into the eradication of the disease.

Members of the MTC cause a granulomatous-caseous-necrotical lesion that mainly affects the lungs and regional lymph nodes but can also extend to other organs such as liver, spleen, kidneys, mammary glands, pericardium, uterus and brain [3]. Their pathogenicity is mainly based on four aspects: (i) their ability to multiply inside the macrophages of the host, (ii) their resistance to phagocytosis, (iii) the chronical inflammatory response elicited in the host leading to the development of granulomas, and (iv) a slow evolution of the infection. TB lesions can be easily confused with those produced by other pathogens such as *Actinomyces* spp., *Rhodococcus equi* or other mycobacteria belonging to the *Mycobacterium avium* complex [4, 5].

Previous studies have suggested the existence of differences in the presentation and evolution of the disease in infected animals, what could be due to the strain involved (characterized by DVR-spoligotyping, MIRU-VNTR typing or whole-genome sequence), owing to its ability to evoke a different immune response [6], and to factors related with host susceptibility [7–9]. Given that the virulence of a pathogen is typically related with its ability to spread [10], the existence of differences in the capacity of MTC strains to cause clinical lesions in infected hosts could have implications in disease eradication given that it could be hypothesized that more virulent strains may be transmitted faster (though could be also detected easier due to the induction of a stronger immune response in the host).

For this reason, we conducted a study to test the hypothesis that the recovery of *M. bovis* isolates belonging to specific spoligotypes could be associated with an increased probability in detecting lesions/severity of such lesions in an infected host, considering a large collection of cattle samples retrieved in the frame of the Spanish eradication program. This sample collection had been assessed for the presence of macroscopical TB-like lesion to evaluate the relationship between the presence and degree of lesions and the spoligotype involved accounting for other possible covariates.

## Methods

### Population of study

A total of 8889 samples from lung and lymph nodes coming from cattle collected from 2011 to 2019 were included in the study. Samples originated from 13 autonomous communities in Spain, and the most represented ones were Madrid (*n* = 3429), Castilla-La Mancha (2966), Aragon (1429) and Valencia (375).

Samples were collected because they originated from animals subjected to slaughter in the frame of the Spanish bovine tuberculosis eradication program (i.e., they were positive in ante-mortem tests). Samples coming from passive surveillance (i.e., abattoir inspection) were discarded to avoid biases (since they were already selected due to the presence of visible lesions).

### Laboratory analysis

The presence of lesions in the samples was established qualitatively (yes/no) and, for samples with lesions, semi quantitatively depending on the type of lesion at the laboratory as previously reported [11]: briefly, in the case of lung samples, a scale from 1 to 5 was used, with:

- Grade 1: small visible lesion of variable size of 1–2 mm.
- Grade 2: five or fewer lesions less than 1 cm.
- Grade 3: more than five lesions less than 1 cm, or one bigger than 1 cm.
- Grade 4: more than one lesion bigger than 1 cm.
- Grade 5: granulomatous lesions along all lung parenchyma.

In the case of lymph nodes (mediastinal, retropharyngeal, tracheobronchial and prescapular), lesions were scored on a 1–3 scale as follows:

- Grade 1: five or less visible lesions of variable size of 1–2 mm.
- Grade 2: more than five lesions less than 1–2 mm, or one bigger than 0.5 cm.
- Grade 3: widely spread lesion affecting all the lymph node.

Samples were then subjected to bacteriological analysis as previously described [12]. Briefly, tissues collected before 2018 coming from the same animal were pooled and homogenized, independently of the presence of lesions but always including lesioned tissues when found, for subsequent decontamination with a hexadecyl pyridinium chloride solution and finally inoculated either in solid Löwenstein-Jensen with 1% sodium pyruvate and Coletsos media (Difco, Madrid, Spain) at 37 °C for up to 12 weeks. From 2018 onwards pooled tissues were decontaminated with N-acetyl-L-cysteine-sodium hydroxide solution (BBL® MycoPrep™) and inoculated into an automated liquid medium (BD BACTEC™ MGIT™) and Löwenstein-Jensen with 1% sodium pyruvate medium at 37 °C for up to 56 days. The presence of *M. bovis* was then confirmed in samples in which bacterial growth was observed by conventional PCR [13]. Finally, the spoligotype and MTC species of the isolated strains were determined by DVR-spoligotyping as previously described [14, 15]. Spoligotype profiles were assigned according to the spoligotype database Mbovis.org [16].

### Statistical analysis

The proportion of samples from that were lesion and/or culture positive was determined and for the latter the most common spoligotypes found was described. Differences in the observed proportion of lesion-positive samples depending on the spoligotype was then assessed using a chi-square test. The diversity of spoligotypes in culture-positive samples was determined through Simpson’s index of diversity (SI) using the package “vegan” [17] in R [18]. Confidence intervals (CI) for the Simpson’s indexes were estimated through 1000 bootstrap replicates using the package “boot” [19]. Then, the severity of the lesions in lesioned samples as determined by the semiquantitative scores given to lesioned lung [1–5] or lymph node [1–3] samples was compared between spoligotypes through a Kruskal-Wallis test followed by a Holm-Bonferroni post-hoc test. Only samples in which spoligotypes were presented in at least 3% of the total population were subjected to the analysis.

Finally, the association between the spoligotype and the presence of lesions in culture-positive samples was evaluated through multivariable mixed logistic regression models. Again, only spoligotypes present in at least 3% of the samples were considered. Models were fitted in a Bayesian framework as:

$$ {logit}^{-1}\left({p}_{ij}\right)={\alpha}_{j\left[i\right]}+{\beta}_1{X}_{i1}+{\beta}_2{X}_{i2}+\cdots +{\beta}_k{X}_{ik} $$

Where *p*_*ij*_ is the probability of finding TB-like lesions in samples from animal *i* from herd *j*, *α*_*j[i]*_ is the random effect for the herd *j*, *β*_*1…*_*β*_*k*_ are the coefficients of the covariables assessed, and *X*_*1…*_*X*_*k*_ the values for the covariables itself.

Covariables available included the autonomous region of origin of the samples and the reason of culling (i.e., positive to intradermotuberculinization [IDTB] or gamma-interferon [IFN-γ]). Herd was included as a random variable to account for the lack of independence of samples with the same origin.

Weakly informative normal N (0, 100) prior distributions were used for the *β* coefficients. The *α* random effect was assumed to follow a normal N(μ, σ^− 2^) distribution, with μ ~ N (0, 1) and σ ~ uniform (0, 10). The best model was selected based on the lowest DIC (Deviance Information Criteria) [20].

Models were fitted in WinBUGS [20] through the R package “R2WinBUGS” [21]. Three Markov Chain Monte Carlo chains were run for 100,000 iterations, with a “burn-in” of the first 25,000 iterations, and posterior distributions were obtained after thinning every 10 iteration. Convergence was assessed visually and more formally using the Gelman-Rubin statistic [22].

## Results

### Descriptive analysis

Macroscopical lesions were detected in approximately one-fourth of the samples included in the study (*n =* 2187/8889; 24.6%). Among the 8889 samples, 2080 (23.4%) were culture-positive and subsequently spoligotyped, leading to the identification of 88 different spoligotypes. Among the culture-positive samples, most presented TB-like lesions (1835/2080; 88.2%). The most common spoligotype retrieved from cattle samples in the collection was SB0121, the most prevalent in the Iberian Peninsula [21]. Overall, spoligotype diversity in the isolate collection was high (SI = 0.871, 95% CI 0.805–0.944), and if only one isolate per herd was considered, the estimated SI did not vary significantly (0.875, 95% CI 0.818–0.936), although the relative proportion of certain spoligotypes was different (data not shown). Overall, seven of the spoligotypes (SB0121, SB0339, SB0134, SB0265, SB0295, SB0120 and SB1142) were isolated in more than 3% of all the samples (and represented 73.8% of all culture-positive samples).

The proportion of lesions in culture-positive samples between spoligotypes was significantly different (*p* ≤ 0.001). SB0120 was the spoligotype in which the highest proportion of lesioned samples was observed (95/98; 96.9%), closely followed by SB1142 (70/74; 94.6%) and SB0295 (98/104; 94.2%), while lesions were less frequent in samples that yielded isolates with the SB0265 (112/129; 86.8%) and SB0339 (351/404; 86.9%) profiles. A total of 1986 lesioned TB target-organ samples from 1600 cattle were evaluated semi-quantitatively depending on the type of lesion. Lesions assessed were found in lungs (*n* = 311), mediastinal (834), retropharyngeal (639), tracheobronchial (150), and prescapular lymph nodes (52) (Additional file 1). The most prevalent type of lesion in lungs was grade 3 (85/311; 27.3%). In the case of the lymph nodes, grade 3 was the most common score (845/1675; 51.0%) (Table 1). Significant differences between the type of lesion observed and the spoligotype present were only found in lungs (*p* = 0.029) and mediastinal lymph nodes (*p* = 0.027), although differences between specific spoligotypes were not detected using post-hoc tests.

**Table 1:** Scores observed in lesioned samples (*n* = 1986) from lung and lymph nodes from cattle

		Target organ											
		Lung		Mediastinal		Retropharyngeal		Tracheobronchial		Prescapular		Total (LN)^a^	
**Type of lesion**	**1**	39	12.7%	250	30.0%	73	11.4%	35	23.3%	13	25.0%	371	22.1%
	**2**	65	20.9%	236	28.3%	167	26.1%	35	23.3%	12	23.1%	450	26.9%
	**3**	85	27.3%	348	41.7%	399	62.4%	80	53.3%	27	51.9%	854	51.0%
	**4**	51	16.4%	–	–	–	–	–	–	–	–	–	–
	**5**	71	22.8%	–	–	–	–	–	–	–	–	–	–
**Total**		311		834		639		150		52		1675	

^a^Lymph nodes

### Multivariable analysis

A total of 1534 samples were included in the multivariable analysis. Animals originated from 423 herds (mean = 3.63 animals per herd, median = 2, range = 1–38). All isolates belonged to seven spoligotypes that had been retrieved in more than 3% of the samples (SB0121, SB0339, SB0134, SB0265, SB0295, SB0120, and SB1142) (1534/2080; 73.8% of all cattle samples). Nearly all (93.9%, 1441/1534) samples came from 4 regions (Madrid, Castilla-La Mancha, Aragon and Valencia), with the remaining regions (Balearic Islands, Andalucia, La Rioja, Extremadura, Navarra, Murcia, Canary Islands and Cataluña) combined in a miscellaneous category (Table 2) (Additional file 2).

**Table 2:** Number of lesioned cattle samples and its association with the spoligotype and region of origin

Variables	N^a^	Lesion		Odds	95% PPI^b^	
		Yes	No		2.5%	97.5%
**Spoligotype**						
SB0121	548	477 (87.1%)	71 (12.9%)	1	–	–
SB0339	404	351 (86.9%)	53 (13.1%)	0.75	0.46	1.18
SB0134	177	154 (87.0%)	23 (13.0%)	1.04	0.59	1.92
SB0265	129	112 (86.8%)	17 (13.2%)	1.03	0.52	2.1
SB0295	104	98 (94.2%)	6 (5.8%)	2.7	1.1	7.1
SB0120	98	95 (96.9%)	3 (3.1%)	6.1	1.96	27.9
SB1142	74	70 (94.6%)	4 (5.4%)	1.88	0.63	7.3
**Region**						
Madrid	596	542 (90.9%)	54 (9.1%)	1	–	–
Castilla-La Mancha	381	338 (88.7%)	43 (11.3%)	0.63	0.36	1.1
Aragon	357	300 (84.0%)	57 (16.0%)	0.41	0.24	0.7
Valencia	107	88 (82.2%)	19 (17.8%)	0.45	0.21	0.91
Others	93	89 (95.7%)	4 (4.3%)	2.25	0.75	8.31
**Total**	1534	1357 (88.5%)	177 (11.5%)	–	–	–

^a^Number of samples. ^b^Posterior probability interval

**Fig. 1:**
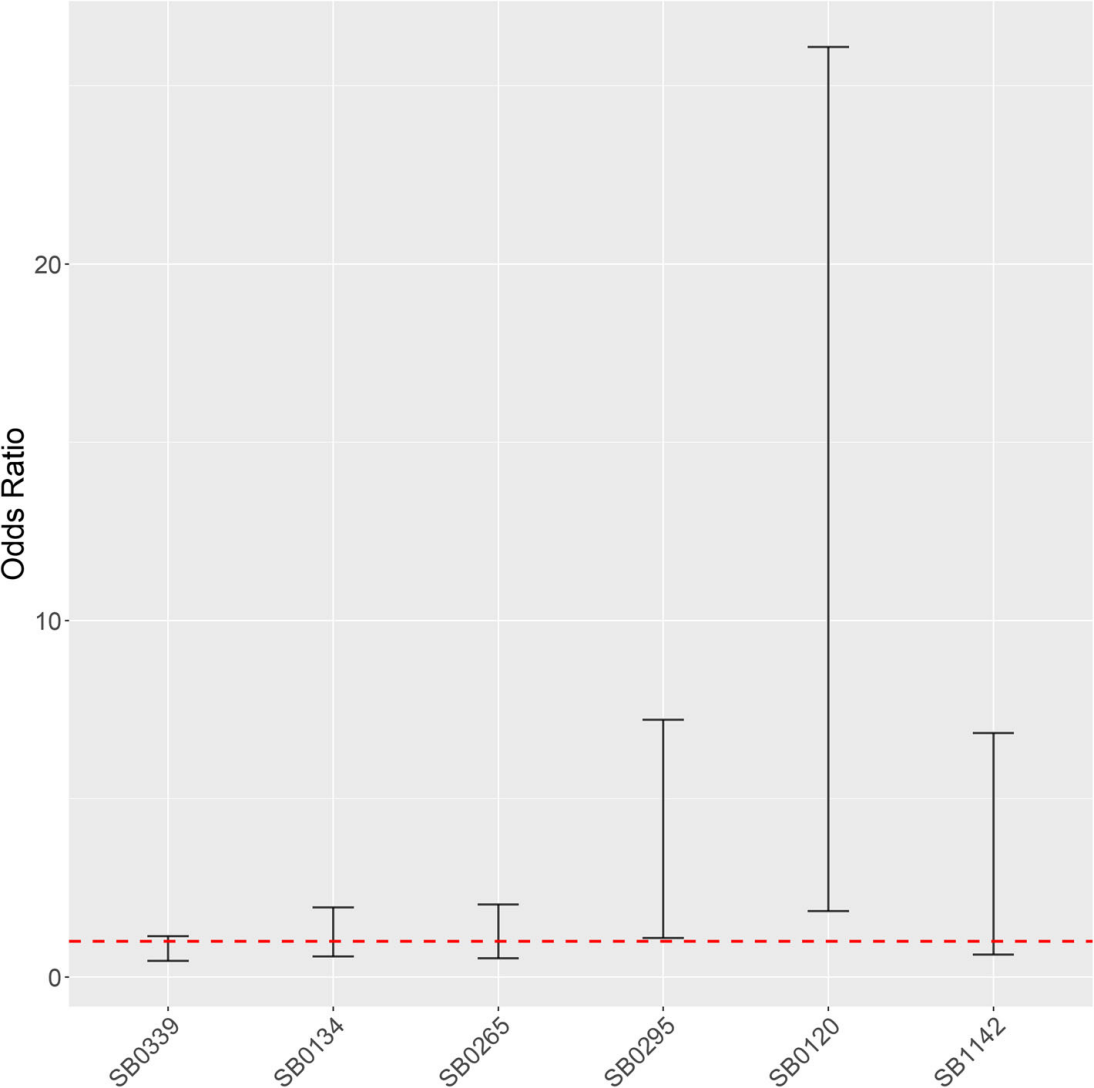
Posterior probability interval of finding a lesion in cattle samples depending on the spoligotype isolated. Spoligotypes were compared with the reference category (SB0121) according to a Bayesian mixed logistic regression model

The final model for cattle included the spoligotype and the region of origin as covariables. Two spoligotypes, SB0120 and SB0295, were associated with an increased probability of finding lesions (Table 2): animals infected with strains belonging to spoligotypes SB0120 and SB0295 had 6.1 (Posterior Probability Interval (PPI) 95% [1.96–27.9]) and 2.7 (PPI 95% [1.1–7.1]) higher odds, respectively, of having a detected lesion compared to the reference (SB0121) (Fig. 1). In addition, an increased risk was observed in samples from animals infected with SB1142 while the opposite was true (i.e., there was a decreased risk) for samples infected with SB0339, although in those cases the 95% PPI included 1 (Table 2).

In addition, samples coming from the regions of Castilla-La Mancha, Aragon and Valencia had lower odds of having lesions than those coming from the reference category (Madrid), although the 95% PPI for samples coming from Castilla-La Mancha included 1 (Table 2).

## Discussion

Animal tuberculosis is one of the most important diseases impacting wildlife and livestock worldwide due to the problems associated to control it, related to its complex epidemiology [23, 24]. Molecular characterization of its causative agent is now performed routinely in many countries (usually through spoligotyping, although whole genome sequencing is increasingly applied) in order to help to identify sources of infection and control disease spread [25, 26]. Differences in the pathogenicity/virulence of MTC strains could have important consequences in disease control, since a more virulent strain may induce the formation of larger lesions in a shorter period (thus increasing transmissibility) [27]. Furthermore, an increased survival in the environment could also provide a competitive advantage to specific strains, since indirect transmission (i.e., due to contact with contaminated environments instead of direct contact with an infected animal) can lead to a significant proportion of new infections in certain areas of Spain [28–30]. However, an increased virulence may also induce a stronger immune response that would be more easily detected in host species subjected to routine diagnostics tests [31], in agreement with previous reports describing a positive correlation between the reaction size of the single intradermal comparative cervical test (SICCT) and the number and size of lesions found [32]. Nevertheless, other studies have found no association between genotype of strains and the outcome of SICCT and therefore this relationship may not (always) hold [33]. Here, we assessed the presence of an association between a certain strain type (as defined by the spoligotype) and the presence and severity of the lesions in a large collection of samples coming from cattle retrieved in the frame of the Spanish TB eradication program [34].

Because of the observational nature of this study, several limitations must be considered when interpreting the results. First, we used the spoligotyping profile as our definition of strain, even though it is determined considering only a small fraction of the genome of *M. bovis* (differences in the Direct Repeat locus, a non-coding region), and thus, distantly related strains may present the same spoligotype while more closely related isolates may have different profiles [35, 36], limiting its discriminatory power, and even more with predominant spoligotypes as in this study. Further studies involving WGS are therefore needed to identify if differences in the spoligotype may be associated with genetic traits that could lead to increased/decreased pathogenicity.

In addition, several variables that could be associated with an increased risk of lesion in infected animals (not related with the specific *M. bovis* strain) and that were not formally included in the analysis could have influenced our results. For example, given the relatively long period (2011–2019) and regions included in the study, there may have been differences in the post-mortem inspection at the abattoir leading to the samples collection analysed here. Nevertheless, given that the presence of lesions/lesion severity was assessed at the same laboratory by the same technicians over time once the samples were collected there should not be differences in the criterium used to score the tissues.

Unfortunately, we had no information of other host-related factors (such as age, breeding type, sex, presence of coinfections, history of tuberculosis in the herd) that could be related with the early/late development of lesions. Also, the fact that only differences in the severity of lesions were seen in organs with a high number of samples could be mainly due to a lack of sample size, impairing our ability to detect significant differences. Nevertheless, accounting for the unit and the region of origin could partially control for the confounding effect of some of these unknown variables. In any case our results must therefore be interpreted carefully, and should be confirmed in animals for which more information is available and, ideally, in experimental conditions.

According to our results, the proportion of culture-positive samples was relatively low (23.4%). This was not unexpected even if all samples originated from animals that were positive to the official TB diagnostic tests (i.e., IDTB and IFN-γ) and were thus presumably infected: bacteriological culture has a limited sensitivity [37], particularly in the early stages of the disease [38]. Since the majority of the cattle population over 6 weeks of age are subjected to regular routine testing, infected animals are typically detected shortly after exposure to pathogen, and in this context ante-mortem tests are expected to outperform bacteriology, which should not be used as a gold-standard to determine the true infectious status of an animal [39].

When only the subset of culture-positive samples was considered, a much higher proportion of lesioned samples was found as expected (88.2%). In turn, most of the lesioned samples were also culture-positive (1755/2187; 80.2%), thus demonstrating the superior performance that culture has to confirm infection in animals in a more advance stage of disease [35, 36].

According to the Bayesian mixed multivariable logistic regression model, a strong effect of the spoligotype was observed, with profiles associated with both high (SB0120, SB0295, SB1142) and low (SB0339) risk of finding lesions in infected animals compared to the reference profile (SB0121) (Table 2). Interestingly, the SB0120 profile (BCG-like spoligotype) had the strongest association with the presence of lesions (Odds = 6.1 [95% PPI 1.96–27.9]). This spoligotype was also considered in a previous study comparing prevalent spoligotypes in cattle in Argentina [40], but in that case it was not associated with increased risk of lesions. However, in that study only SB0120 out of the seven spoligotypes evaluated here was considered. Furthermore, samples considered in this study originated from cattle identified as infected during abattoir inspection, a population discarded here.

Regarding the findings related to the severity of TB lesions according to a semiquantitative score, samples infected with SB0120 did not have higher scores than those with other spoligotypes in which higher proportions of mediastinal lymph nodes and lung samples reached the maximum degree severity (Additional file 3): most of the lesions due to SB0120 in these organs were of grade one out of three (mediastinal lymph node, 23/53; 43.4%) and grade three out of five (lungs, 7/17; 41.2%). Interestingly, the other spoligotype with an increased risk of detection of lesions (SB0295) was associated with an increased severity in lesions found in both mediastinal lymph nodes and lung (Additional file 3), although significant differences between specific spoligotypes were not confirmed in the post-hoc analysis.

The relative frequency of the spoligotypes found in samples was determined including more than one isolate from a given epidemiological unit (i.e., herd) and thus could be affected by oversampling of certain units infected with specific spoligotypes. Therefore, it should not be considered as a representative sample of the true underlying diversity in the target populations sampled. Still, when only one isolate per unit was considered a similar diversity and proportion of spoligotypes was observed, suggesting that this was not a major source of bias in our results. Furthermore, the inclusion of epidemiological unit in the multivariable model corrected for the lack of independence between observations coming from the same herd.

## Conclusions

This preliminary analysis suggests that the infection by *M. bovis* strains may lead to the occurrence of lesions preferentially when certain strains (spoligotypes) are involved. Therefore, in-depth studies to determine how the strain can influence disease progression accounting for host-related factors are needed to further understand the epidemiology of the infection, especially with regards to the immune response triggered by it, a key factor for early diagnosis.

## Supplementary Information

**Additional file 1.** Scored lesioned lymph node samples.

**Additional file 2.** Distribution of the most isolated spoligotypes in cattle within different regions from Spain.

**Additional file 3.** Frequency of isolates belonging to the most frequent spoligotypes cultured from lesioned mediastinal lymph nodes and lungs from cattle depending on the type of lesion found.

## Data Availability

The datasets used and/or analysed during the current study are available from the corresponding author on reasonable request.

## Abbreviations

*IDTB*: *Intradermotuberculinization*
IFN-γ: Gamma-interferon
MTC: *Mycobacterium tuberculosis* complex
PPI: Posterior probability interval
SI: Simpson’s diversity index
SICCT: Single intradermal comparative cervical test
TB: Animal tuberculosis
