# Supplementary material for: Spoligotype-specific risk of finding lesions in tissues from cattle infected by Mycobacterium bovis
Source: BMC Vet Res. 2021 Apr 7;17:148. doi: 10.1186/s12917-021-02848-3 (PMC8028093; doi:10.1186/s12917-021-02848-3)
Supplement: Supplementary file 1 — Additional file 1. Scored lesioned lymph node samples. [file 12917_2021_2848_MOESM1_ESM.pdf]

Additional file 1

Spoligotype-Specific Risk Of Finding Lesions In Tissues From Cattle Infected By *Mycobacterium bovis*

Alberto Gómez-Buendía[1], Beatriz Romero[1], Javier Bezos[1,2], Francisco Lozano[1], Lucía de Juan[1,2], Julio Álvarez[1,2*]

[1] VISAVET Health Surveillance Centre, Universidad Complutense de Madrid, Spain

[2] Departamento de Sanidad Animal, Facultad de Veterinaria, Universidad Complutense de Madrid, Spain

*Correspondence: jalvarez@visavet.ucm.es

Scored lesioned lymph node samples

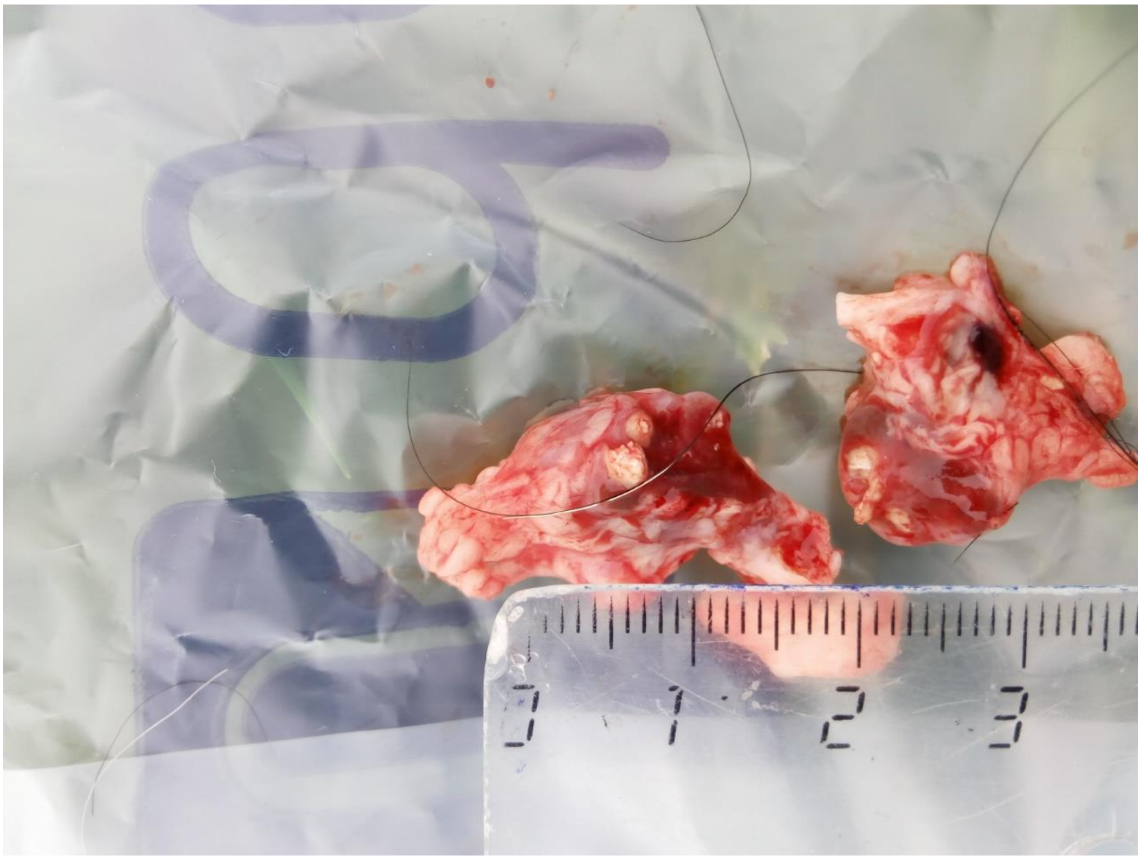

Figure 1. Grade 1 TB lesion

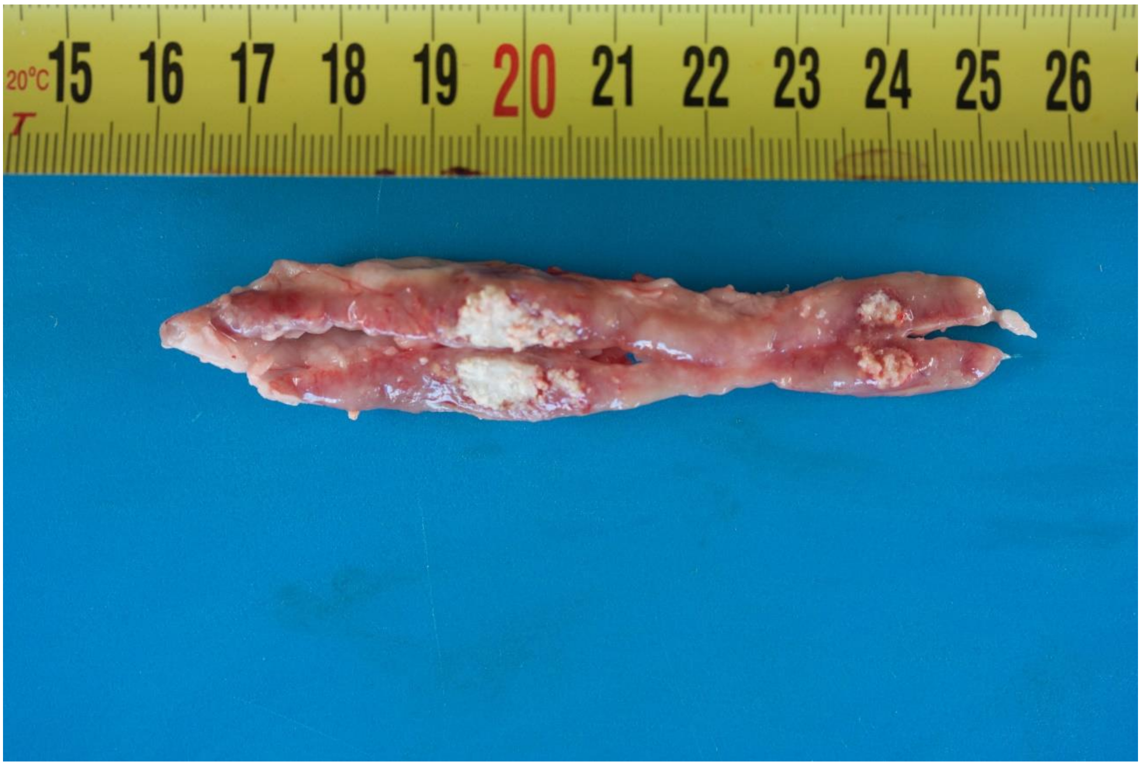

Figure 2. Grade 2 TB lesion

Additional file 1. *Gómez-Buendía et al.*

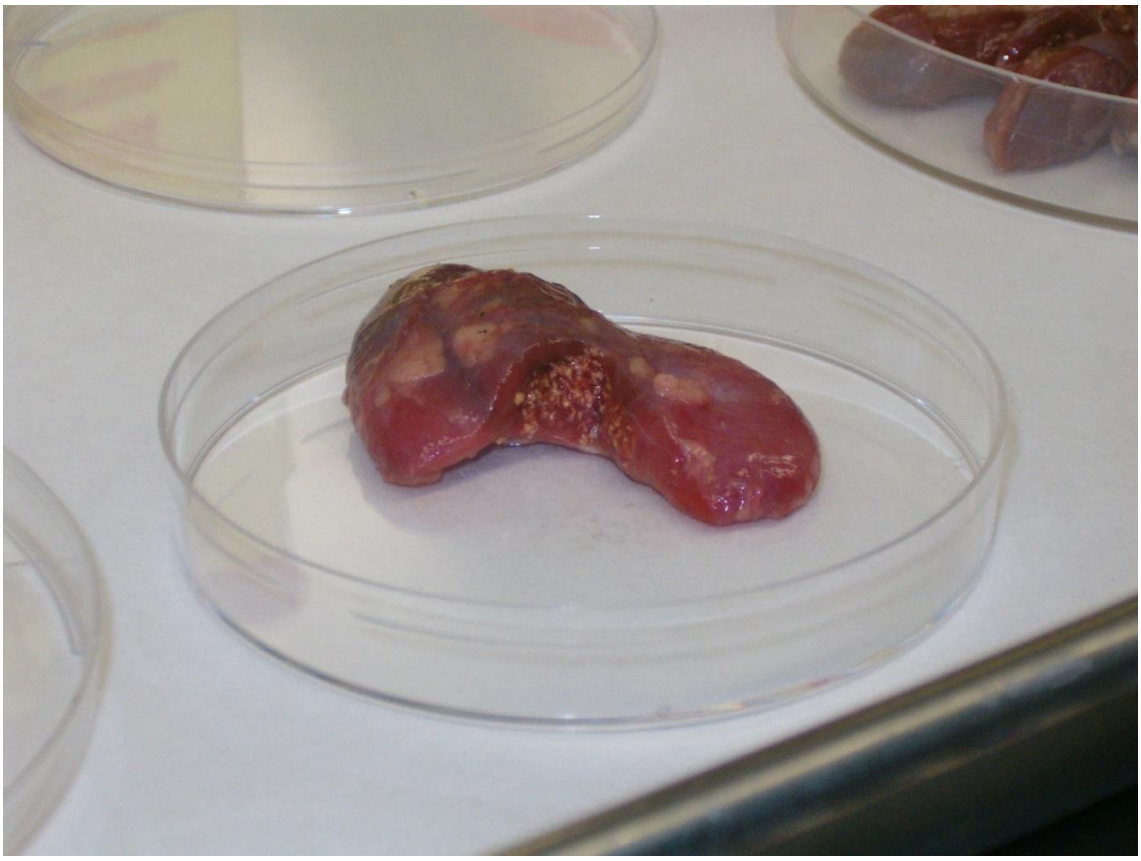

Figure 3. Grade 2 TB lesion

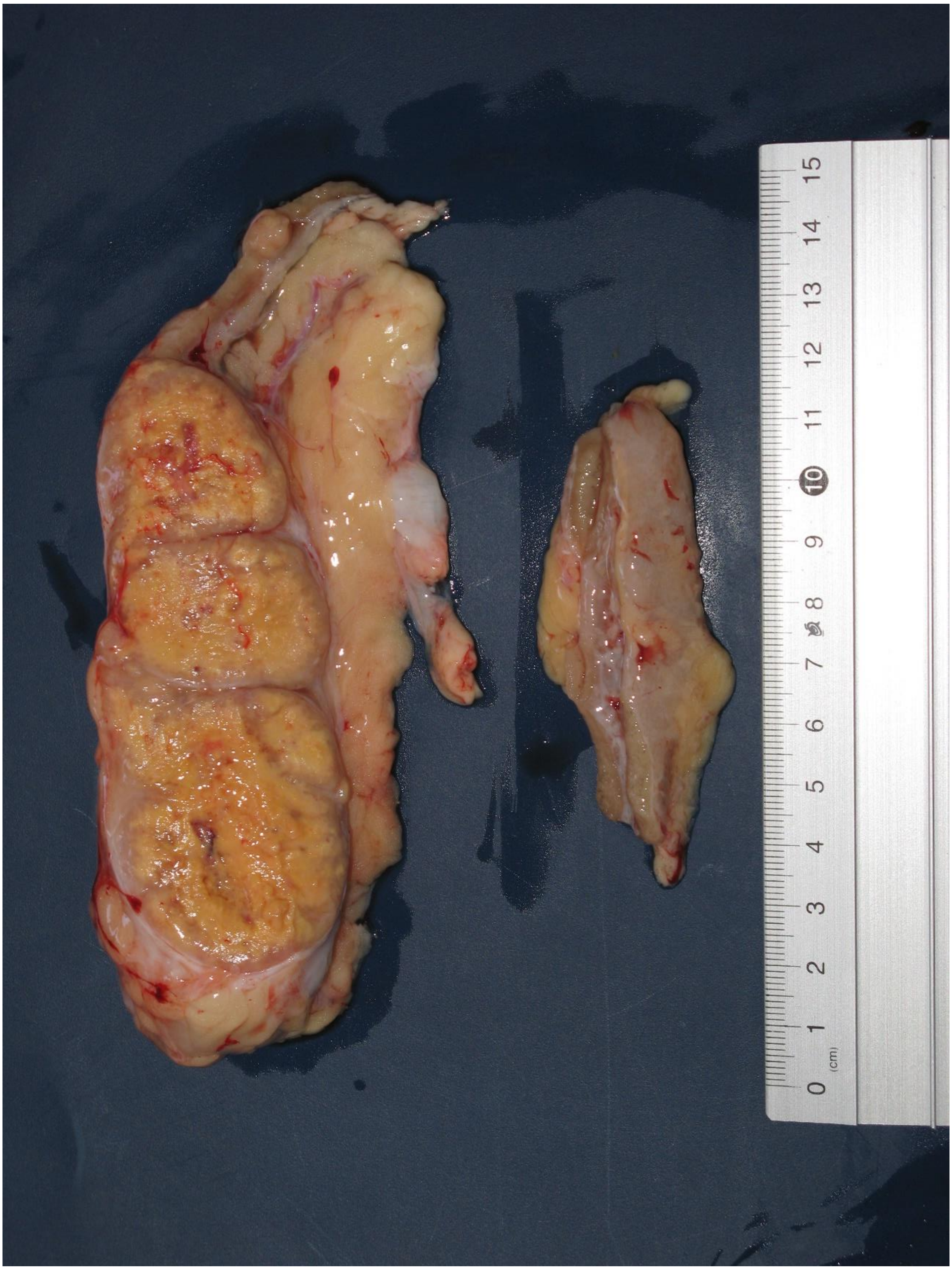

Figure 4. Grade 3 TB lesion

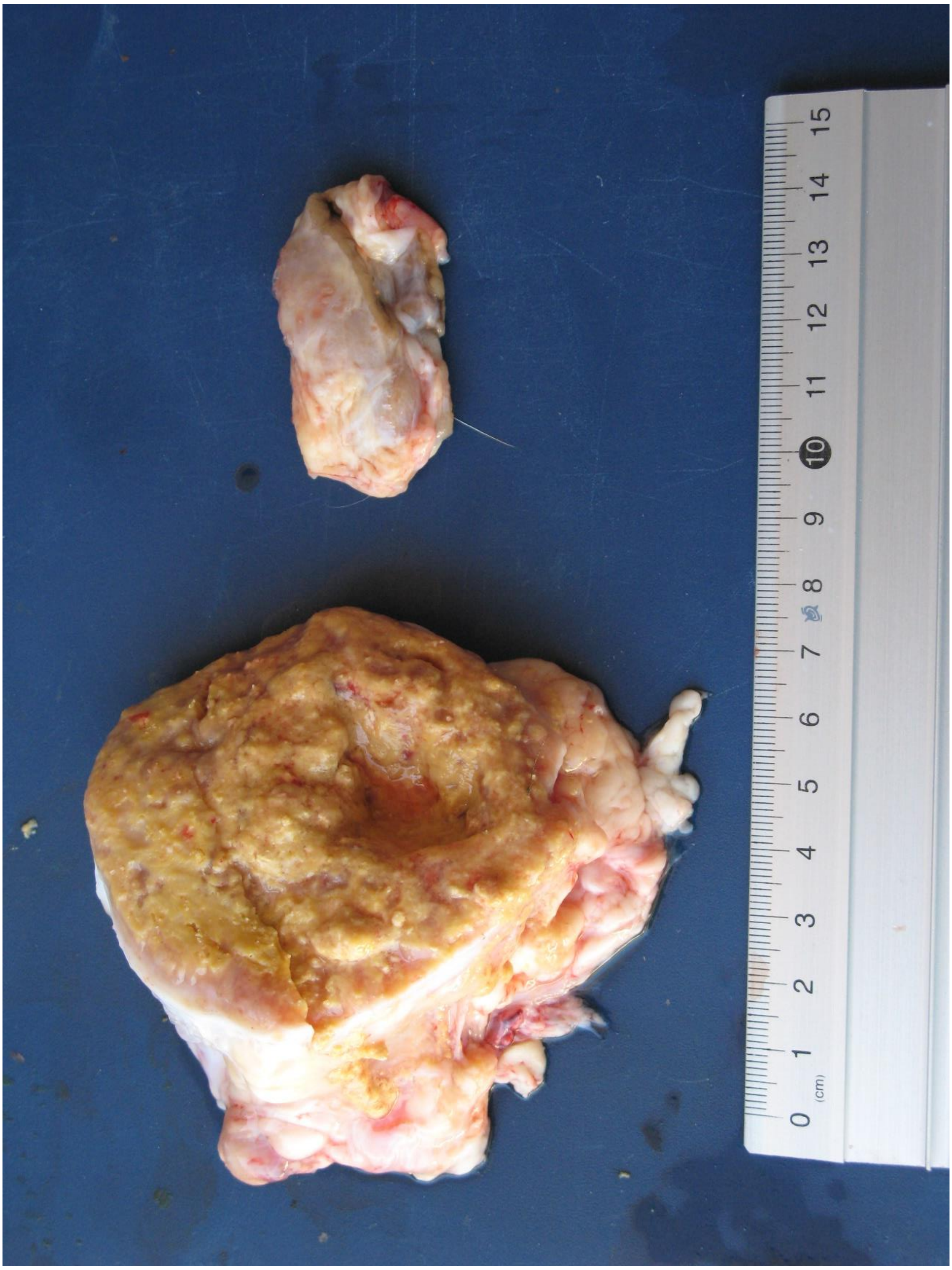

Figure 5. Grade 3 TB lesion
