# Supplementary material for: Spoligotype-specific risk of finding lesions in tissues from cattle infected by Mycobacterium bovis
Source: BMC Vet Res. 2021 Apr 7;17:148. doi: 10.1186/s12917-021-02848-3 (PMC8028093; doi:10.1186/s12917-021-02848-3)
Supplement: Supplementary file 2 — Additional file 2. Distribution of the most isolated spoligotypes in cattle within different regions from Spain. [file 12917_2021_2848_MOESM2_ESM.pdf]

Additional file 2

Spoligotype-Specific Risk Of Finding Lesions In Tissues From Cattle Infected By *Mycobacterium bovis*

Alberto Gómez-Buendía[1], Beatriz Romero[1], Javier Bezos[1,2], Francisco Lozano[1], Lucía de Juan[1,2], Julio Álvarez[1,2*]

[1] VISAVET Health Surveillance Centre, Universidad Complutense de Madrid, Spain

[2] Departamento de Sanidad Animal, Facultad de Veterinaria, Universidad Complutense de Madrid, Spain

*Correspondence: jalvarez@visavet.ucm.es

Distribution of the most isolated spoligotypes in cattle within different regions from Spain

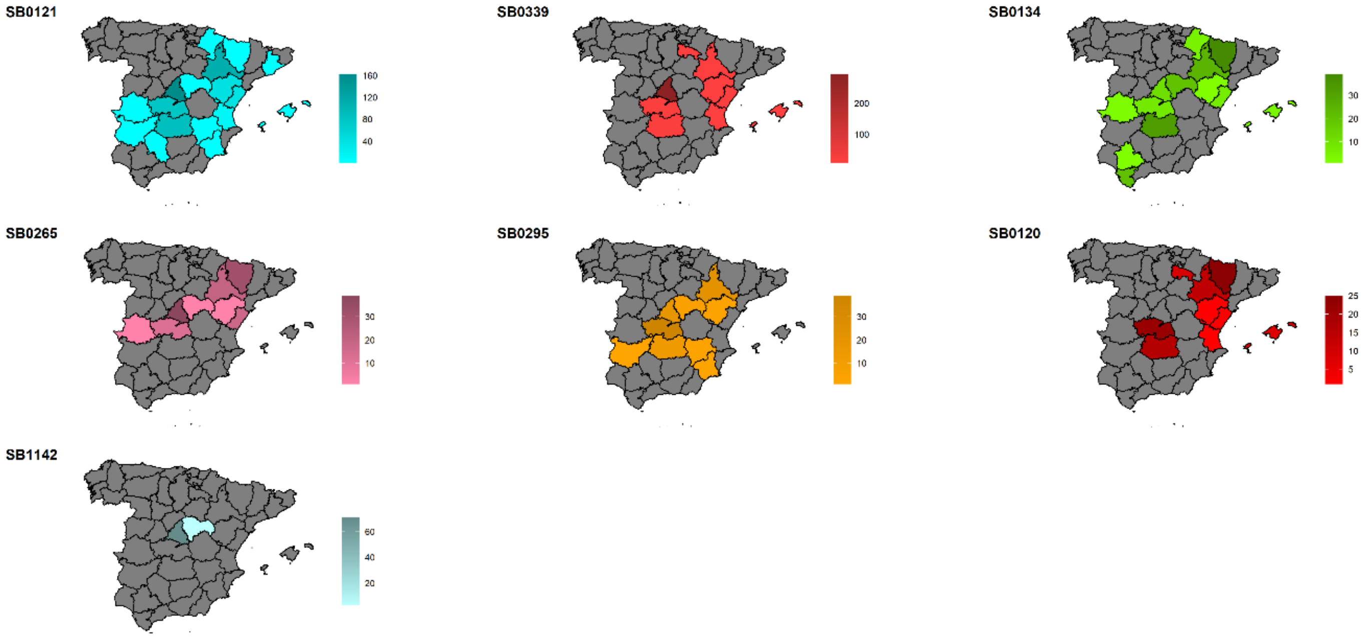

Additional file 2 *Gómez-Buendía et al.*
