# Supplementary material for: Spoligotype-specific risk of finding lesions in tissues from cattle infected by Mycobacterium bovis
Source: BMC Vet Res. 2021 Apr 7;17:148. doi: 10.1186/s12917-021-02848-3 (PMC8028093; doi:10.1186/s12917-021-02848-3)
Supplement: Supplementary file 3 — Additional file 3. Frequency of isolates belonging to the most frequent spoligotypes cultured from lesioned mediastinal lymph nodes and lungs from cattle depending on the type of lesion found. [file 12917_2021_2848_MOESM3_ESM.pdf]

Additional file 3

Spoligotype-Specific Risk Of Finding Lesions In Tissues From Cattle Infected By *Mycobacterium bovis*

Alberto Gómez-Buendía[1], Beatriz Romero[1], Javier Bezos[1,2], Francisco Lozano[1], Lucía de Juan[1,2], Julio Álvarez[1,2*]

[1] VISAVET Health Surveillance Centre, Universidad Complutense de Madrid, Spain

[2] Departamento de Sanidad Animal, Facultad de Veterinaria, Universidad Complutense de Madrid, Spain

*Correspondence: jalvarez@visavet.ucm.es

Frequency of isolates belonging to the most frequent spoligotypes cultured from cattle from lesioned mediastinal lymph nodes from cattle depending on the type of lesion found

	Type of lesion			
Spoligotype	1	2	3	Total
SB0121	83 (32.4%)	61 (23.8%)	112 (43.6%)	256
SB0339	49 (34.8%)	37 (27.4%)	51 (37.8%)	135
SB0134	19 (27.1%)	23 (32.9%)	28 (40.0%)	70
SB0265	19 (30.7%)	18 (29.0%)	25 (40.3%)	62
SB0120	23 (43.4%)	13 (24.5%)	17 (32.1%)	53
SB0295	7 (14.9%)	10 (21.3%)	30 (63.8%)	47
SB1142	9 (29.0%)	9 (29.0%)	13 (42.0%)	31
Total	207 (31.7%)	171 (26.2%)	276 (42.2%)	654

Frequency of isolates belonging to the most frequent spoligotypes cultured from cattle from lesioned lungs from cattle depending on the type of lesion found

	Type of lesion					
Spoligotype	1	2	3	4	5	Total
SB0121	17 (19.3%)	25 (28.4%)	18 (20.5%)	10 (11.4%)	18 (20.5%)	88
SB0339	6 (13.0%)	8 (17.4%)	18 (39.1%)	7 (15.2%)	7 (15.2%)	46
SB0134	2 (6,3%)	3 (9.4%)	8 (25.0%)	9 (28.1%)	10 (31.3%)	32
SB0265	1 (4.6%)	5 (22.7%)	6 (27.3%)	5 (22.7%)	5 (22.7%)	22
SB0120	2 (11.8%)	1 (5.9%)	7 (41.2%)	5 (29.4%)	2 (11.8%)	17
SB0295	0 (0.0%)	2 (20.0%)	3 (30.0%)	1 (10.0%)	4 (40.0%)	10
SB1142	2 (20.0%)	3 (30.0%)	3 (30.0%)	0 (0.0%)	2 (20.0%)	10
Total	30 (13.3%)	47 (20.9%)	63 (28.0%)	36 (16.0%)	49 (21.8%)	225
